# Development of Optimal Digesting Conditions for Microplastic Analysis in Dried Seaweed *Gracilaria fisheri*

**DOI:** 10.3390/foods10092118

**Published:** 2021-09-08

**Authors:** Rizky Prihandari, Weeraya Karnpanit, Suwapat Kittibunchakul, Varongsiri Kemsawasd

**Affiliations:** Institute of Nutrition, Mahidol University, Salaya, Nakhon Pathom 73170, Thailand; rizky.pri@mahidol.student.ac.th (R.P.); weeraya.kar@mahidol.ac.th (W.K.); suwapat.kit@mahidol.ac.th (S.K.)

**Keywords:** microplastics, *Gracilaria fisheri*, enzymatic digestion, oxidative digestion, Raman spectroscopy, vegetal tissue

## Abstract

Currently, research on the accumulation of microplastics (MPs) in the marine food web is being highlighted. An accurate and reliable digestion method to extract and isolate MPs from complex food matrices has seldom been validated. This study aimed to compare the efficacy of MP isolation among enzymatic-, oxidative-, and the combination of two digestion methods on red seaweed, *Gracilaria fisheri*. The dried seaweed sample was digested using three different methods under various conditions using enzymes (cellulase and protease), 30% H_2_O_2_, and a combination of enzymes and 30% H_2_O_2_. The method possessing the best digestion efficiency and polymer recovery rate of MPs was selected, and its effect on spiked plastic polymer integrity was analyzed by Raman spectroscopy. As a result, the enzymatic method rendered moderate digestion efficiency (59.3–63.7%) and high polymer recovery rate (94.7–98.9%). The oxidative method using 30% H_2_O_2_ showed high digestion efficiency (93.0–96.3%) and high polymer recovery rate (>98%). The combination method was the most effective method in terms of digestion efficiency, polymer recovery rate, and expenditure of digestion time. The method also showed no chemical changes in the spiked plastic polymers (PE, PP, PS, PVC, and PET) after the digestion process. All the spiked plastic polymers were identifiable using Raman spectroscopy.

## 1. Introduction

Annual plastic production reached 381 million tonnes in 2015, which was around 200-fold higher than the total amount of plastic produced in the 1950s [1]. The increased use and disposal of plastics coupled with their low degradation rate result in the accumulation of plastic waste in the environment [1]. Environmental stressors such as UV radiation, elevated temperature, oxidation, and water abrasion degrade this waste into small-sized plastic particles (smaller than 5 mm), so-called microplastics (MPs) [2,3,4]. The abundance of MPs in terrestrial [5] and aquatic [6] ecosystems and the findings of MP contamination on various food products [7,8] have aroused global concern about MP accumulation in the food web.

MPs can be accumulated in a wide variety of organisms [9,10,11] and transferred up the trophic levels of the food chain through ingestion [12,13]. Nelms et al. (2018) demonstrated that trophic transfer was the major MP ingestion pathway for any species whose feeding ecology involves the consumption of whole prey, including humans [14]. The European Food Safety Authority (EFSA) announced the importance of studies on MP contamination in commercial food products [15] and the impact of MPs on human health [16]. A recent study has revealed that the human body is capable of eliminating MPs with a particle size range of 50–500 μm via feces [17]. Nevertheless, MPs smaller than 20 μm can be accumulated in the organs and cause adverse effects [18,19] while the larger particles can induce oxidative stress, thus leading to chronic inflammation in the human body [20].

Over the last few years, attempts to detect and analyze MPs in food items have been made to assess and manage the risks associated with MP exposure. In MP analysis, the digestion of food matrices is crucial to the dissolution of organic matter and the isolation of synthetic polymers from the food samples. A lack of standard digestion methods for MP analysis results in the difficulty to compare the effectiveness of digestion performance. The quality of different digestion methods can be assessed through the ability to reduce the matrix complexity, i.e., digestion efficiency and to prevent plastic polymer degradation, which is usually demonstrated as polymer recovery rate [21].

To isolate MPs from marine animals, previous research applied different digestion methods including acid [8,22], alkaline [23], oxidative [11,23,24,25], enzymatic [23,26,27,28], and a combination of several methods, such as a stepwise method using sodium hydroxide (NaOH) and nitric acid (HNO_3_) [29]. Meanwhile, the extraction of MPs from vegetal tissues, namely nori seaweed, broccoli, lettuce, carrot, and potato, is limited to using 65% HNO_3_ [30] and a more extensive digestion method using a combination of cellulase, protease, and 30% hydrogen peroxide (H_2_O_2_) [31]. To the best of our knowledge, however, a study of digestion methods for MP analysis in *Gracilaria fisheri* (*G. fisheri*) has not yet been conducted.

Among chemical digestions, HNO_3_ obtained a higher digestion efficiency than potassium hydroxide (KOH) and H_2_O_2_ in prawn and mussel, respectively [10,32]. However, the use of acid likely caused several defects in a wide range of plastic polymers, including high-density polyethylene (HDPE), low-density polyethylene (LDPE), polyethylene terephthalate (PET), polystyrene (PS), polyamide (PA), and polypropylene (PP) [10,33]. For alkaline digestion, increasing the temperature up to 60 °C with NaOH and KOH accelerated the digestion efficiency in fish up to 91% and 98%, respectively [21]. However, that condition degraded polyethylene (PE), polycarbonate (PC), and PET [22]. Oxidative digestion (30% H_2_O_2_) was more efficient than the use of alkaline solution to digest biogenic matter in marine sediment [34], especially when the elevated temperature (55–65 °C) was applied [22]. However, the increase in H_2_O_2_ concentration and temperature caused a destructive effect, especially on synthetic PA [35]. Enzymatic digestion was categorized as a mild digesting approach, in which different enzymes possess different digestion efficiency. For example, the use of different proteolytic enzymes in mussel obtained digestion efficiency results ranging from 78 to 88% [27]. However, the enzymatic digestion did not cause physical changes on polyvinyl chloride (PVC), PP, PE, PS, PET, and PA [27]. For combination digestion, the use of optimized protocols, combination of proteinase-K and sodium perchlorate (NaClO_4_), obtained high digestion efficiency (>97%) and did not cause any destructive effect on PS, PE, PVC, nylon, and polyester [36].

*G. fisheri*, an edible red seaweed naturally distributed in the shoreline area, is widely used as an ingredient in commercial food products [37]. Moreover, it may be used as a future herbal medicine due to its anti-tumor activity [38] and as a feed supplementation due to its anti-viral activity [39]. The roles of *G. fisheri* in treating cholangiocarcinoma in human and white spot syndrome in shrimp are obtained from sulfated galactan, which is a polysaccharide found in the cell walls of marine algae [38,39]. The thallus is mainly structured by complex polysaccharides (60.7%, mostly cellulose), protein (11.6%), and minerals [40], while the main components of other aquatic organisms like bivalves are protein and lipid [41]. *G. fisheri* and other varieties of seaweed are becoming important indicators for assessing MP contamination in the marine environment as they can potentially trap MPs from the surrounding water [42,43]. Owing to its complex structure, the optimization of digestion conditions for MP analysis in *G. fisheri* is still a challenging issue in the field of MP research.

This study aimed to compare the effectiveness of different digestion methods in various conditions for isolating MPs from dried *G. fisheri*. Three digestion methods, including enzymatic-, oxidative- and combination of enzymatic and oxidative methods, were studied for the digestion efficiency, polymer recovery rate, and microscopic observation under a stereomicroscope. In addition, the chemical composition of each spiked plastic polymer after the digestion process using an optimal method in suitable conditions was further analyzed by using Raman spectroscopy.

## 2. Materials and Methods

### 2.1. Sample Preparation

Dried *G. fisheri* collected from the coast of the Gulf of Thailand was resized into 2–5 mm lengths. The sample was stored in a sealed aluminium foil bag prior to use. The analysis of the moisture content of dried *G. fisheri* was carried out according to the Association of Official Analytical Chemists (AOAC) method no 930.04 [44].

### 2.2. Contamination Control

MP contamination control was applied throughout the entire analytical procedure (sample preparation, digestion, and analysis) to avoid contamination from workers, environment, and equipment. All work surfaces were thoroughly cleaned using 70% ethanol to prevent contamination [45]. A negative control was processed in parallel with each digestion experiment as the procedural blank for quality assurance.

### 2.3. Optimization of Digestion Method

#### 2.3.1. Enzymatic Method

Dried *G. fisheri* (0.25 g on a dry weight basis; DW) was transferred to a 250 mL Erlenmeyer flask containing 25 mL of sodium phosphate buffer saline (PBS, pH 5.0). The flask was incubated at 50 °C, 90 rpm for 30 min prior to simultaneous addition of cellulase (15,000 IU/mL, Reach Biotechnology, Bangkok, Thailand) and protease (≥80,000 IU/mL, Reach Biotechnology, Bangkok, Thailand). The flask was then further incubated at 50 °C, 90 rpm for 30 h. The concentration of cellulase used was varied as follows: 1%, 3%, and 5% (*v*/*v*), while the concentration of protease used was 5% (*v*/*v*) in all enzymatic conditions.

#### 2.3.2. Oxidative Method

Two different weight by volume ratios of sample to 30% H_2_O_2_ (Merck, Darmstadt, Germany) at 1:50 (OD50) and 1:100 (OD100) were studied. The 30% H_2_O_2_ was added to each 0.25 g (DW) of dried *G. fisheri*. Then, the mixture was incubated at 60 °C, 90 rpm for 96 h. 

#### 2.3.3. Combination Method

*G. fisheri* was digested by using the enzymatic and oxidative methods in sequential order. Briefly, 0.25 g (DW) of dried *G. fisheri* was digested by using 1% (*v*/*v*) cellulase and 5% (*v*/*v*) protease at 50 °C, 90 rpm for 2 h. Then, the enzyme was inactivated at 85 °C for 10 min. Subsequently, 30% H_2_O_2_ was added with the sample to H_2_O_2_ ratio of 1:100 and was incubated at 60 °C, 90 rpm for 36 h.

### 2.4. Monitoring of Enzymatic Digestion

Enzymatic digestates (600 μL) collected at different time points (0 to 30 h) were placed in a water bath at 85 °C for 10 min to inactivate enzymes. Then, the solutions were rapidly cooled on ice for 5 min prior to centrifugation at 1000× *g* for 10 min. The supernatants were collected and stored at 4 °C for subsequent 3,5-dinitrosalicylic acid (DNS) and 2,4,6-trinitrobenzene sulfonic acid (TNBS) assays.

#### 2.4.1. DNS Assay

The DNS assay described by Miller (1959) was performed to monitor the enzymatic hydrolysis of carbohydrates through measuring the released reducing sugars [46] The DNS solution was prepared by dissolving 0.1 g of 98% 3,5-dinitrosalicylic acid (Loba Chemie, Mumbai, India) in 2 mL of 2 N NaOH (Qrec, Auckland, New Zealand). Then, 3 g of sodium potassium tartrate (Kemaus, Cherrybrook, Australia) was added to the solution at 50 °C. The solution was subsequently adjusted to 10 mL with deionized water. An aliquot of the sample (100 μL) was mixed with DNS reagent (100 μL). The mixture was incubated in a water bath at 95 °C for 10 min and was cooled down before adding 500 μL of deionized water. Then, the absorbance was read at 540 nm using a spectrophotometer (Spectra max plus 384, Molecular Devices, Ramsey, MN, USA), and the reducing sugar concentration was expressed in terms of D-glucose (MP Biomedicals, Strasbourg, France).

#### 2.4.2. TNBS Assay

The TNBS assay described by Benjakul and Morrissey (1997) was performed to monitor the enzymatic hydrolysis of protein through measuring the released α-amino acids [47]. The 0.01% TNBS reagent was prepared by diluting the 5% TNBS stock solution (Sigma-Aldrich, Saint Louis, MO, USA) with 0.2 M phosphate buffer (pH 8.2). An aliquot of the sample (25 μL) was mixed with 400 μL of the buffer. Then, 200 μL of 0.01% TNBS reagent was added. The mixture was incubated in a water bath at 50 °C for 30 min, and the reaction was terminated by adding 400 μL of 0.1 M sodium sulfite. The absorbance was read at 420 nm using the spectrophotometer, and free α-amino acid concentration was expressed in terms of L-leucine (Sigma-Aldrich, Saint Louis, MO, USA).

### 2.5. Digestion Efficiency and Polymer Recovery Rate

After digestion, the sample was centrifuged at 1000× *g* for 10 min. The supernatant was then collected and filtered through a 2.5 μm Whatman™ filter paper (Buckinghamshire, United Kingdom) using a vacuum pump (300, Rocker, New Taipei City, Taiwan) with a pressure of approximately 20 to 40 kPa. The filter paper with retained digestate from the seaweed sample was dried to constant weight at 70 °C. The weight of the digested sample on the filter paper was used to calculate the digestion efficiency using the following equation [21].

(1) $$\mathrm{Digestion}\;\mathrm{Efficiency}\;\left(\%\right)=\frac{W_{i}-\left(W_{a}-W_{b}\right)}{W_{i}}\;x\;100$$

where W_i_ = Initial weight of seaweed sample; W_a_ = Weight of dry filter paper after filtration; W_b_ = Weight of dry filter paper before filtration.

For the polymer recovery rate, five types of plastic polymers, including medium-density polyethylene (PE) (powder, Sigma-Aldrich, Saint Louis, MO, USA), polypropylene (PP) (granular, Sigma-Aldrich, Saint Louis, MO, USA), polystyrene (PS) (beat, Sigma-Aldrich, Saint Louis, MO, USA), polyethylene terephthalate (PET) (granular, Sigma-Aldrich, Saint Louis, MO, USA), and polyvinyl chloride (PVC) (powder, Sigma-Aldrich, Saint Louis, MO, USA), were analyzed as the control spikes in parallel with all the digestion conditions. The positive control contained 2 mg of each type of plastic polymer and 1 pellet of PET. The total weight of the five spiked plastic particles on the filter paper was used to calculate the polymer recovery rate using the following equation [21].

(2) $$\mathrm{Polymer}\;\mathrm{Recovery}\;\mathrm{Rate}\;\left(\%\right)=\frac{W_{a}-W_{b}}{W_{i}}\times 100$$

where W_i_ = Initial weight of spiked plastic particles; W_a_ = Weight of dry filter paper after filtration; W_b_ = Weight of dry filter paper before filtration.

To evaluate the effect of each digestion method, the digestion efficiency and polymer recovery rate obtained from each digesting condition were substracted with those obtained from negative controls. To determine the effect of the digestion procedures on the plastic polymers, the obtained digestates and spiked plastic particles retained on the surface of the dried filter paper were visualized by using a stereomicroscope (Stemi 305, Carl Zeiss Microscopy GmbH, Göttingen, Germany) at 10× and 40× objectives with ZEN 3.0 Blue edition software.

### 2.6. Polymer Characterization

In this study, the spiked plastic particles that underwent the combination digestion method were characterized by Raman spectroscopy to investigate the effect of the digestion process on the integrity of each polymer. The Raman spectroscopy system (XploRA Plus, Horiba, Kyoto, Japan) was equipped with 500 μm confocal hole, 100 μm slit, and 600 grooves/mm grating. Then 785 nm radiation of laser and a 50× objective were applied. Raman spectra were recorded in the wavenumber range of 200–3500 cm^−1^, with an acquisition time of 20 s repeated three times. The equipment was calibrated with silicon wafer prior to use. The Raman system was operated using LabSpec6 software. After the detection process, the spectra of the spiked plastic polymers were compared with the reference plastic spectra in the KnowItAll spectral library (Horiba France SAS, Palaiseau, France).

### 2.7. Statistical Analysis

All experiments were conducted at least in triplicate. The results were expressed as mean ± standard deviation when appropriate. Statistical analysis was performed using Analysis of Variance (ANOVA) with Duncan’s Multiple Range test. The *p* < 0.05 was considered to be statistically significant. The data were analyzed using the IBM SPSS Statistics for Windows version 26.0 software (IBM Corp., Armonk, New York, NY, USA).

## 3. Results

### 3.1. Enzymatic Method

Three concentrations of cellulase (1, 3, and 5%, *v*/*v*) in combination with 5%, *v*/*v*, protease were tested. Enzymatic digestion was performed at 50 °C with shaking. The DNS and TNBS assays were applied to monitor the hydrolysis of *G. fisheri* by cellulase and protease through the analysis of reducing sugars and free amino groups, respectively.

**Figure 1:**
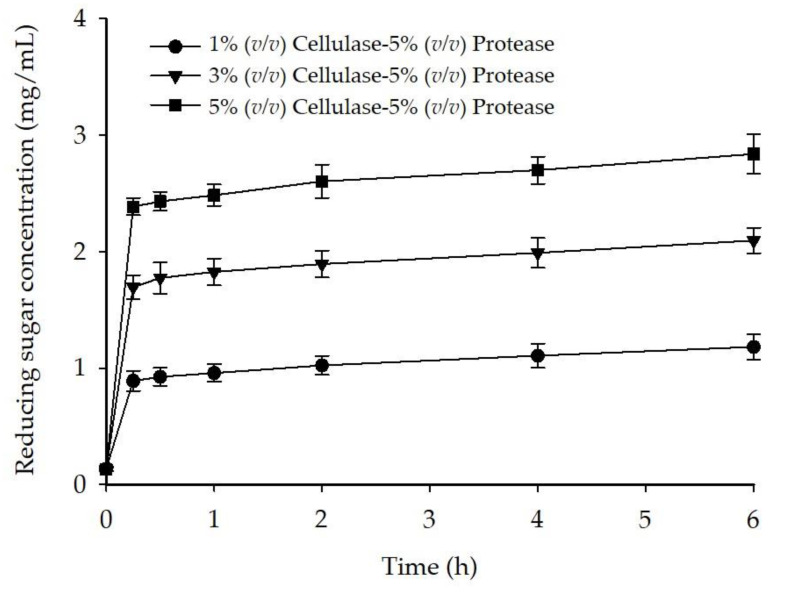
Time course of reducing sugar release during the enzymatic digestion of *G. fisheri*.

Based on the released reducing sugar, a similar hydrolysis profile of *G. fisheri* cellulose was observed for all enzymatic conditions (Figure 1). Overall, the degradation of *G. fisheri* cellulose rapidly took place during the first 15 min of digestion, which corresponded to a sharp increase in reducing sugar concentration due to glucose liberation. The initial rate of reducing sugar released significantly increased (*p* < 0.05) with increasing cellulase concentration, and the rates obtained with 3% and 5% (*v*/*v*) cellulase were 2-fold and 3-fold higher than the rate obtained with 1% (*v*/*v*) cellulase, respectively. After 15 min of digestion, the cellulose hydrolysis reached a plateau where no significant increase (*p* > 0.05) in reducing sugar concentration over time was observed. At the end of observation at 6 h, reducing sugar concentrations obtained with 1%, 3%, and 5% (*v*/*v*) cellulase (1.18 mg/mL, 2.10 mg/mL, and 2.84 mg/mL, respectively) were significantly different (*p* < 0.05).

**Figure 2:**
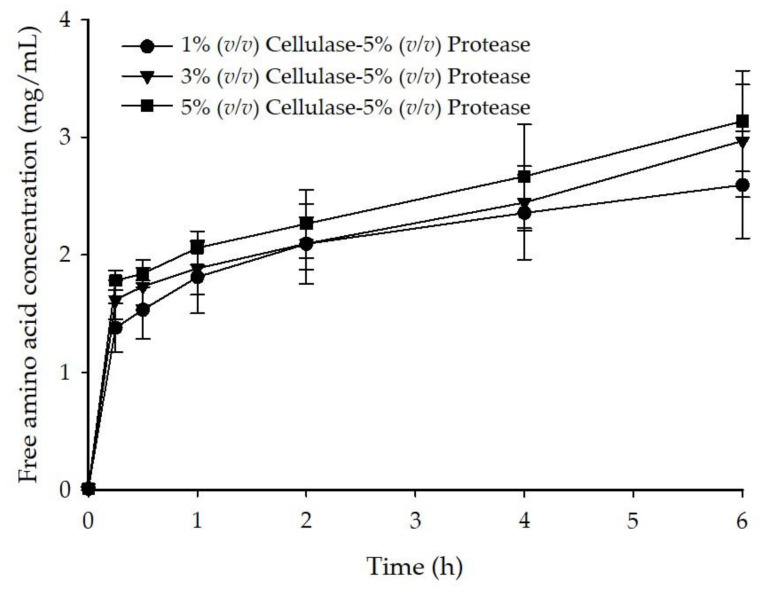
Time course of free amino group release during the enzymatic digestion of *G. fisheri*.

Similar to the results of cellulose hydrolysis, all tested enzymatic conditions resulted in a resemble hydrolysis profile of *G. fisheri* protein based on the release of free amino acids (Figure 2). As a whole, a rapid degradation of *G. fisheri* protein occurred during the first 15 min of digestion as it was shown by a marked increase in free amino acid concentration, while the release of free amino acids was retarded after 15 min of digestion. As expected, the concentration of free amino acids did not significantly increase (*p* > 0.05) when the digestion was prolonged from 4 h to 6 h; hence, this suggested that the hydrolysis reaction was likely to reach a plateau after 6 h of digestion. At the end of observation at 6 h, the free amino acid concentrations obtained with 1%, 3%, and 5% (*v*/*v*) cellulase combined with 5% (*v*/*v*) protease were 2.60 mg/mL, 2.97 mg/mL, and 3.13 mg/mL, respectively. It was noted that the higher cellulase concentration seemed to promote the hydrolysis activity of protease.

The digestion efficiency and polymer recovery rate observed after 30 h for enzymatic digestion of *G. fisheri* are shown in Table 1. Results suggested that the highest digestion efficiency (63.7%) was obtained when using 5% (*v*/*v*) cellulase and 5% (*v*/*v*) protease. This finding was in agreement with the results obtained from DNS and TNBS assays, which showed that an increased cellulase concentration had a more pronounced effect on the degradation of *G. fisheri*. Conversely, the digestion with 5% (*v*/*v*) cellulase and 5% (*v*/*v*) protease resulted in the lowest recovery rate of the spiked plastic particles (94.7%), while the use of 1% (*v*/*v*) cellulase and 5% (*v*/*v*) protease rendered the highest polymer recovery rate (98.9%).

### 3.2. Oxidative Method

For the oxidative method, two samples at 30% H_2_O_2_ ratios, including OD50 and OD100, were tested to determine the digestion efficiency over 96 h of digestion. As shown in Table 2, *G. fisheri* was digested almost completely at 6 h of oxidative digestion at which the digestion efficiencies observed for OD50 and OD100 were about 93.0% and 93.4%, respectively. The efficiencies of OD50 and OD100 reached over 95% after 48 h and 24 h of digestion, respectively, whereas the increase in digestion time from 48 h to 72 h did not result in any significant improvement (*p* > 0.05) in digestion efficiency. Surprisingly, the efficiencies of both OD50 and OD100 significantly decreased (*p* < 0.05) when extending the digestion time to 96 h.

**Figure 3:**
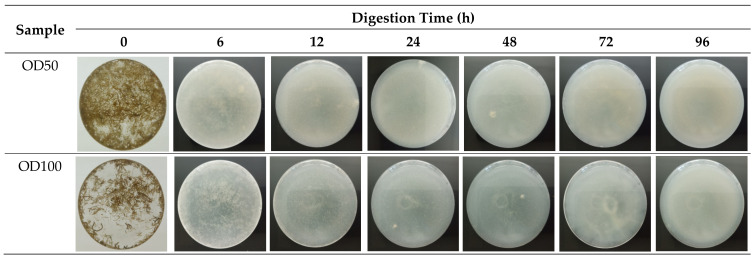
Appearance of dried G. fisheri after oxidative digestion. Bottom view of the digestate in a 250 mL Erlenmeyer flask.

For the polymer recovery rate of the oxidative method, there was no significant effect of 30% H_2_O_2_ and digestion duration on the rate of polymer recovery, and oxidative digestion with all-time intervals conferred high recovery rates above the value of 95%, which was set by Karami et al. (2017) [21]. However, there were significant differences in the appearance of undigested organic and inorganic materials in solutions as well as on the filter papers when these were visually assessed (Figure 3). Between 24 h and 48 h of digestion, the undigested seaweed fragments could be observed by the naked eye and at 72 h, no seaweed fragment was observed. Meanwhile, the prolongation of digestion time from 72 h to 96 h led the solution to become more turbid which could be due to the suspended solids or dissolved matter.

**Figure 4:**
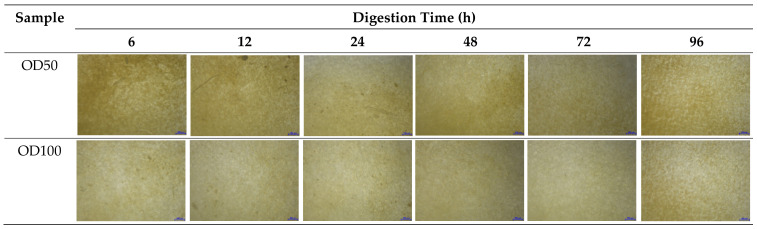
Microscopic observation of dried *G. fisheri* after oxidative digestion. Specimens were viewed on filter papers at 10× objective.

A noticeable amount of retained seaweed on the filter paper was clearly observed for OD50 treatment. This might interfere with the further analysis of MP visual identification under the stereomicroscope (Figure 4). For both oxidative treatments, the filter papers exhibited a deep yellow color, of which the color intensity for OD50 treatment seemed higher.

### 3.3. Combination Method

The combination method involves two steps, of which the 2 h of enzymatic digestion using 1% (*v*/*v*) cellulase and 5% (*v*/*v*) was performed in the first step, followed by the 36 h of oxidative digestion using the ratio of sample to 30% H_2_O_2_ of 1:100. The enzymatic digestion condition was selected based on the best polymer recovery rate (98.9%), while the oxidative digestion condition was selected based on the best digestion efficiency and microscopic observation results. As shown in Table 3, the combined use of enzymatic and oxidative methods could improve both digestion efficiency and polymer recovery rate up to 97.4% and 99.7%, respectively, within a total 38 h of digestion. This method was considerably faster compared to the oxidative method, which took 48 h to 72 h to obtain a digestion efficiency of about 96%. Compared to the enzymatic method, the combination method was more efficient in obtaining complete digestion of *G. fisheri*.

The different digesting methods caused assorted effects on the five types of spiked plastic polymers in positive controls, in which a good microscopic appearance of each particle was observed in the combination method. On the other hand, the dark background color due to the increased enzyme concentration disrupted the microscopic observation for small and transparent particles which were PE, PS, and PVC, while the application of 30% H_2_O_2_ caused a defect on PET (see Supplementary Materials Figure S1).

**Figure 5:**
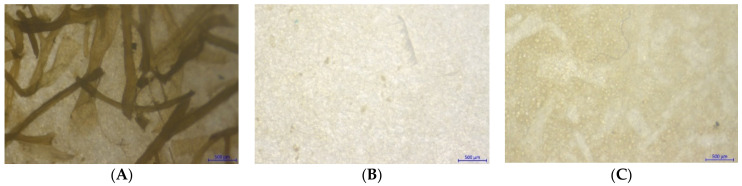
Dried *G. fisheri* and suspected MPs after enzymatic digestion 1% (*v*/*v*) cellulase and 5% (*v*/*v*) protease (**A**), oxidative digestion using sample to 30% H_2_O_2_ ratio of 1:100 (**B**), and combination digestion using 1% (*v*/*v*) cellulase and 5% (*v*/*v*) protease and oxidative ratio of 1:100 (**C**). Specimens were viewed at 10× objective. Rectangular sign points at suspected MPs.

**Figure 6:**
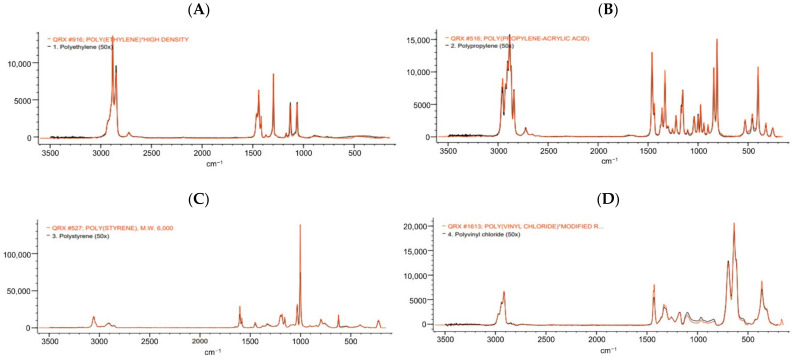

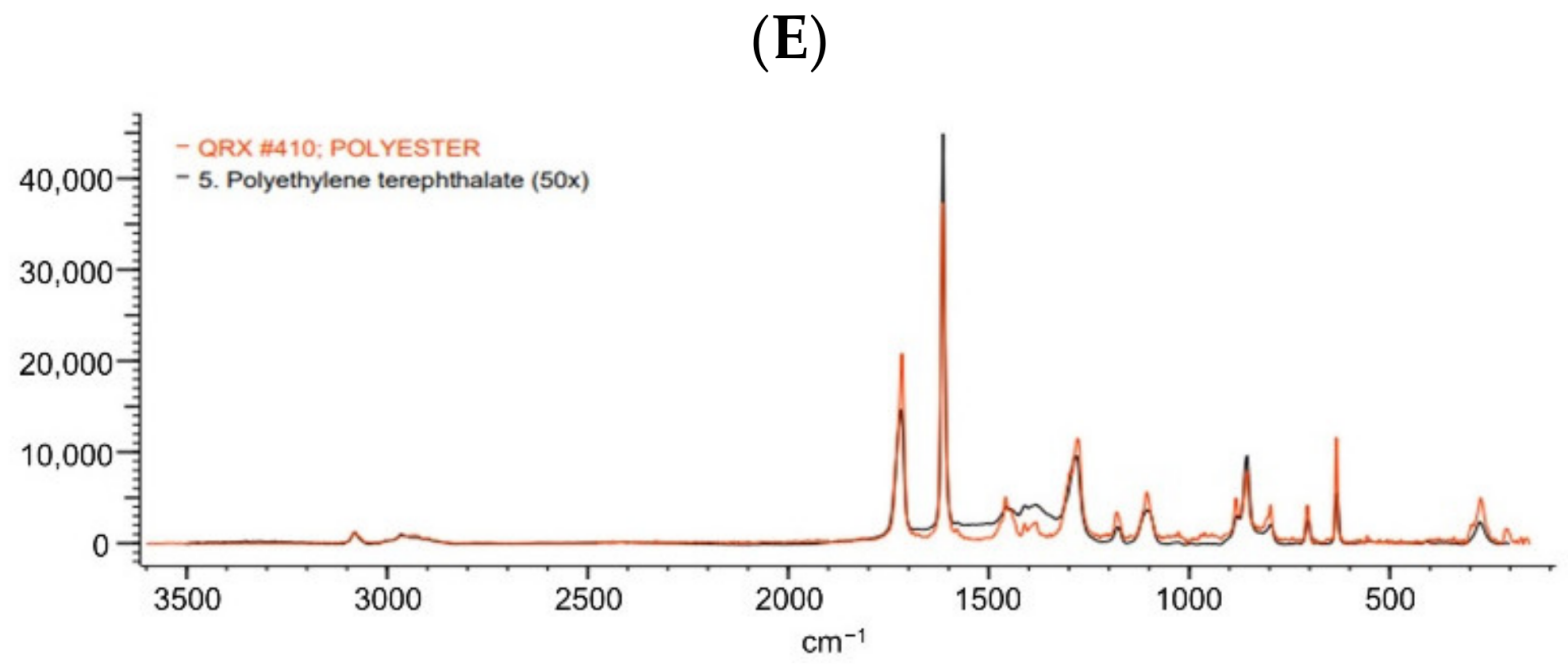
Raman spectra of polyethylene (**A**), polypropylene (**B**), polystyrene (**C**), polyvinyl chloride (**D**), and polyethylene terephthalate or polyester (**E**). The spectra taken for spiked plastic polymers that underwent the combined enzymatic–oxidative digestion are shown in black, while the reference spectra are shown in red.

The profound microscopic observation on samples suggested that the combination method promoted a good visualization of suspected MP particles under a stereomicroscope as a suspected small fiber could be seen clearly without any interference from the digested seaweed sample (Figure 5C). The Raman spectra of all spiked plastic polymers that underwent enzymatic-oxidative digestion matched their reference spectra (Figure 6). This finding revealed that the combination method could degrade the matrix of the seaweed sample without altering the chemical characteristics of the spiked plastic polymers and hence, the polymer recovery rate could be significantly improved through the use of a combination method with a shorter digestion duration.

## 4. Discussion

Digestion is the most important process for isolating MPs in living organisms where MPs might adhere to the organism tissues [22]. Many attempts have been made to develop efficient digestion methods with a focus on the improvement of organic matter destruction and polymer recovery for analyzing MPs in various foods [9,21,26,28,48]. However, the standard digestion protocol for MP investigation has not yet been established. This study, thus, focused on the optimization of the digestion method and its condition for MP analysis in *G. fisheri*, which is an important aquatic organism for human consumption.

Enzymatic approaches are known to possess several advantages over chemical methods such as mild process conditions and high specificity. However, the major limitations of the enzymatic method are the cost and time required [49]. In the present study, the increase in cellulase concentration from 1% to 5% (*v*/*v*) combined with 5% (*v*/*v*) protease led to the improvement of digestion efficiency from 59.3% to 63.7% and the increase of released reducing sugar. The improvement of cellulose hydrolysis was correlated to the increase of cellulase concentration as reported by Kumari et al. (2020) [50]. However, these results indicated incomplete digestion, which might be caused by the restricted substrate specificity of the enzyme [51]. Apart from cellulose, *G. fisheri* also contains hemicellulose and lignin [52] which are resistant to digestion by cellulase.

The polymer recovery rate obtained using the enzymatic method was considered a high recovery rate, categorized based on the value, between 95% to 105%, which was set by Karami et al. (2017) [21]. In this study, an increase in enzyme concentration led to a slightly lower polymer recovery rate. This might be explained by the hydrolysis of PET in that protease can hydrolyze the ester bonds of the general molecular structure of PET [53]. However, the destructive effect was not observed through polymer visualization under a stereomicroscope. According to previous studies, the enzymatic method did not cause any adverse effect on plastic polymers [26,27,54].

Briefly, this study showed that 30% H_2_O_2_ is one of the most versatile oxidative and bleaching agents. H_2_O_2_ can digest a wide range of carbohydrates, proteins, and lipids as the result of the high digestion efficiency and polymer recovery rate as observed in many food materials [21,24]. In previous studies, H_2_O_2_ at a high concentration (30–35%) was used to digest mussel and fish samples for MP analysis [9,11,21,24]. The success of the oxidative method on the biogenic matter in animal and plant tissues was also previously reported by Nuelle et al. (2014) [34]. However, a long period of digestion is required. A complete oxidative digestion in marine bivalves was obtained within 3 days [24], while fish tissue was partially digested for 7 days which might correspond to the low temperature and sample to H_2_O_2_ ratio used in the digestion [9]. In this study, an extended time from 72 h to 96 h showed a decrease in digestion efficiency. It was similar to the study of Prata et al. (2019) on natural organic matter which applied oxidative digestion using 30% H_2_O_2_ at 50 °C and reported a decrease of digestion efficiency of about 10.2% from 1 h to 6 h, but gave no explanation of this finding [55]. Meanwhile, the increase in color intensity in sample OD50 might be explained by the accumulation of undigested samples. The increase of sample amount commonly needs prolongation of the digestion time [24]. Many studies on the optimization of digestion methods recommend the use of H_2_O_2_ in different food matrices. For example, the study of Prata et al. (2019) showed an improvement in digestion efficiency and shortened the digestion time by using 30% H_2_O_2_ with a catalyst (Fe(II)) [55]. The limitations of the H_2_O_2_ digestion are the need for high temperature and time consumption [9,24,34], besides affecting polymer degradation and the toxicity toward human health [10,21,35,56].

For the combination method, the enzymatic digestion was performed prior to oxidative digestion to avoid the disruption of protease and cellulase activities which could be influenced by the changes in pH value. According to the chemical data, 30% H_2_O_2_ has pH value ≤ 3.5 [57], while the optimal pH for cellulase and protease is 5.0 [58,59]. A previous study of Abdulhameed et al. (2005) reported that the enzyme was sensitive to instability due to the pH changes under or over the optimum range [60]. The combination method provided the best results of both digestion efficiency and polymer recovery rate. Moreover, the clear microstructures of MPs obtained by microscopic observation emphasized a good performance of the combination method. These results suggested that the combination method was the most optimal digestion method for *G. fisheri*. Mintenig et al. (2017) and Hurley et al. (2018) also reported the effectivity of enzyme–oxidative combination for digesting the organic matter for MP analysis [35,61].

For polymer characterization, Raman spectroscopy is widely known due to its ability at a low limit of detection and wide spectral range [62]. In this study, the chemical composition of five spiked polymers (PE, PP, PS, PVC, and PET) that underwent the combination method of digestion was confirmed based on their Raman spectral fingerprints. Given Raman spectra of spiked PP, PS, and PVC, an absorption peak was obtained in the region of 2780–2980 cm^−1^. This range corresponded to C–H vibration [63]. Given the Raman spectra of spiked PE and PET, an absorption peak was obtained in the region of 1580–1640 cm^−1^. This spectral range corresponded to aromatic bending vibration [63]. The absorption peaks of five spiked plastic polymers matched with the peaks of reference for the plastic polymers. This indicated no significant effect on the chemical composition of the spiked plastic polymers after the digestion process even on the sensitive polymers, such as PE, PS, and PET. Likewise, our results were in agreement with Mbachu et al. (2021) and Löder et al. (2017) who reported no destructive effect on PE, PP, PS, PVC, and PET after sequential enzymatic and oxidative digestion processes [64,65]. Overall, the combined enzymatic–oxidative digestion method optimized in this study could be used effectively for isolating MPs from *G. fisheri* seaweed and exhibited a high potential to be employed as the sample preparation method for MP analysis in other polysaccharide and protein-rich materials. Moreover, the time expenditure of this combination method was shorter than the previously reported protocol, which required 74 h for the digestion of nori seaweed [31].

## 5. Conclusions

For research on MP analysis in marine organisms, diverse analytical digestion methods have been tailored for specific food matrices to achieve the removal of biogenic matter. This study highlighted the optimization of the digestion conditions for MP analysis in the red seaweed, *G. fisheri*. Significant effects of the enzymatic and oxidative methods, as well as the combination method on *G. fisheri*, were identified. The high polysaccharide content of algae is one of the most common obstacles in the isolation of MPs from the sample. The enzymatic method using cellulase and protease resulted in moderate digestion efficiency. Meanwhile, the oxidative approach afforded great compatibility with the seaweed digestion but it required a long digestion period. The combined use of enzymatic and oxidative digestion methods successfully destroyed the dried *G. fisheri* tissue and offered high MP retention. This method is suggested as a promising digestion protocol for MP analysis in *G. fisheri* and might be able to be extensively applied to other vegetal tissues for MP analysis.

## Figures and Tables

**Table 1:** Digestion efficiency and polymer recovery rate observed for the enzymatic method for *G. fisheri*.

Digestion Condition	Digestion Efficiency (%)	Polymer Recovery Rate (%)
1% (*v*/*v*) cellulase and 5% (*v*/*v*) protease	59.3 ± 2.0 ^a^	98.9 ± 0.5 ^b^
3% (*v*/*v*) cellulase and 5% (*v*/*v*) protease	61.6 ± 1.3 ^b^	95.3 ± 1.3 ^a^
5% (*v*/*v*) cellulase and 5% (*v*/*v*) protease	63.7 ± 1.2 ^c^	94.7 ± 0.8 ^a^

Enzymatic digestion was performed at 50 °C, 90 rpm for 30 h. Data are expressed as mean ± SD of at least triplicate experiments. The values with different lower case letters in a column are significantly different (*p* < 0.05).

**Table 2:** Digestion efficiency and polymer recovery rate observed for oxidative digestion of *G. fisheri*.

Sample	Digestion Time (h)					
	6	12	24	48	72	96
**Digestion Efficiency (%)**						
OD50	93.0 ± 0.3 ^aA^	93.5 ± 0.4 ^aAB^	93.9 ± 0.7 ^aB^	95.0 ± 0.2 ^aC^	95.2 ± 0.3 ^aC^	94.1 ± 0.3 ^aB^
OD100	93.4 ± 0.3 ^aA^	93.8 ± 0.3 ^aAB^	95.3 ± 0.5 ^bC^	95.8 ± 0.3 ^bCD^	96.3 ± 0.2 ^bD^	94.2 ± 0.8 ^aB^
**Polymer Recovery Rate (%)**						
Positive control	98.7 ± 0.9 ^A^	99.1 ± 2.9 ^A^	98.9 ± 10.8 ^A^	98.6 ± 0.6 ^A^	98.3 ± 3.1 ^A^	97.6 ± 3.6 ^A^

Data are expressed as mean ± SD of at least triplicate experiments. The values with different lower-case letters in a column and capital letters in a row are significantly different (*p* < 0.05).

**Table 3:** The comparison of digestion efficiency, polymer recovery rate, and digestion time for the optimized enzymatic-, oxidative-, and combination methods.

Digestion Method	Digestion Efficiency (%)	Polymer Recovery Rate (%)	Digestion Time (h)
Enzymatic method 1% (*v*/*v*) cellulase and 5% (*v*/*v*) protease	59.3 ± 2.0 ^a^	98.8 ± 0.5 ^a^	30
Oxidative method OD100	96.3 ± 0.2 ^b^	98.3 ± 3.1 ^a^	72
Combination method	97.4 ± 0.5 ^b^	99.7 ± 0.1 ^a^	38

Data are expressed as mean ± SD of at least triplicate experiments. The values with different letters in the column are significantly different (*p* < 0.05).

## Data Availability

All data are contained within the article and Supplementary Materials.

## Supplementary Materials

The following are available online at https://www.mdpi.com/article/10.3390/foods10092118/s1, Figure S1: Microscopic appearance of reference and spiked plastic particles incubated with different digesting conditions.
