# Supplementary material for: Development of Optimal Digesting Conditions for Microplastic Analysis in Dried Seaweed Gracilaria fisheri
Source: Foods. 2021 Sep 8;10(9):2118. doi: 10.3390/foods10092118 (PMC8467988; doi:10.3390/foods10092118)
Supplement: Supplementary file 1 [file foods-10-02118-s001.zip › foods-1339377-supplementary.pdf]

Supplementary Information

Development of Optimal Digesting Conditions for Microplastics Analysis in Dried Seaweed *Gracilaria fisheri*

Rizky Prihandari, Weeraya Karnpanit, Suwapat Kittibunchakul and Varongsiri Kemsawasd,*

Institute of Nutrition, Mahidol University, Salaya, Nakhon Pathom, Thailand 73170;
rizky.pri@mahidol.student.ac.th (R.P.); weeraya.kar@mahidol.ac.th (W.K.);
suwapat.kit@mahidol.ac.th (S.K.)
* Corresponding author: varongsiri.kem@mahidol.ac.th(V.K.)

a)

Initial condition of reference plastic particles	Final condition after digestion process of spiked plastic particles				
	Enzymatic Method			Oxidative Method	Combination Method
	1% Cellulase+5% Protease	3% Cellulase+5% Protease	5% Cellulase+5% Protease		

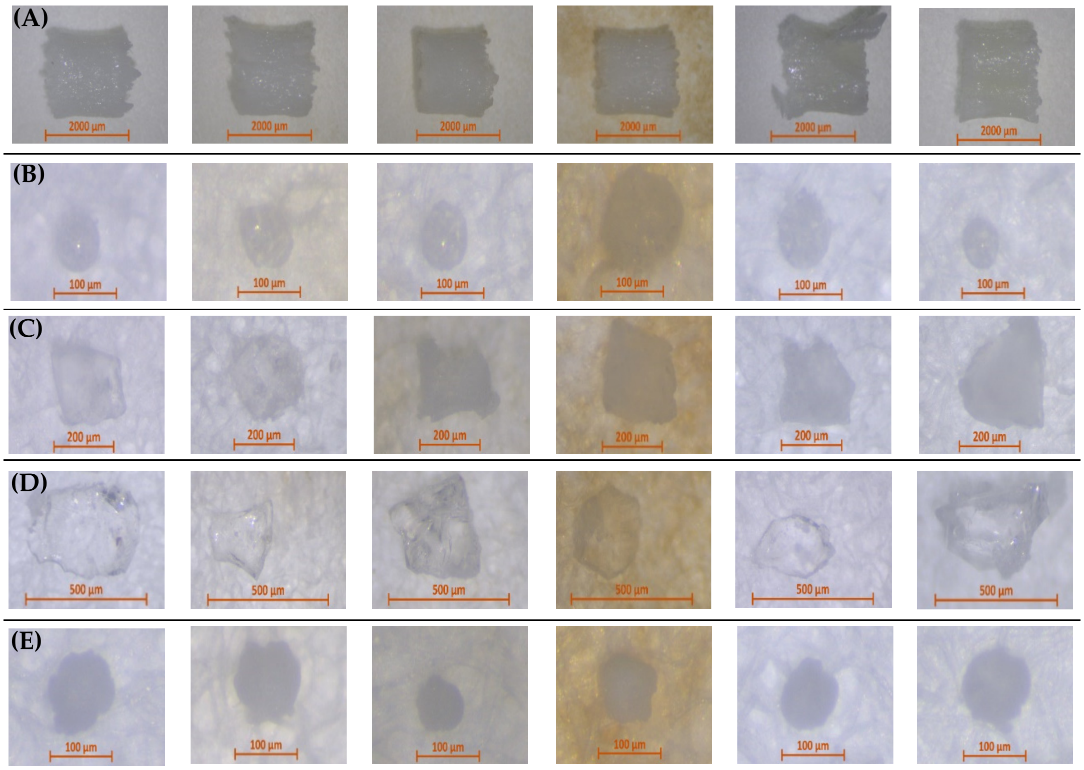

Figure S1: Microscopic appearance of reference and spiked plastic particles incubated with different digesting conditions

*Figure S1: Microscopic appearance of reference and spiked plastic particles incubated with enzymatic, oxidative, and combination methods i.e., polyethylene terephthalate (**A**), polyethylene (**B**), polypropylene (**C**), polystyrene (**D**), and polyvinyl chloride (**E**). All plastic particles were observed by using 10x and 40x objectives.*
